# Estimation of the National Disease Burden of Influenza-Associated Severe Acute Respiratory Illness in Kenya and Guatemala: A Novel Methodology

**DOI:** 10.1371/journal.pone.0056882

**Published:** 2013-02-27

**Authors:** James A. Fuller, Aimee Summers, Mark A. Katz, Kim A. Lindblade, Henry Njuguna, Wences Arvelo, Sammy Khagayi, Gideon Emukule, Nivaldo Linares-Perez, John McCracken, D. James Nokes, Mwanajuma Ngama, Sidi Kazungu, Joshua A. Mott, Sonja J. Olsen, Marc-Alain Widdowson, Daniel R. Feikin

**Affiliations:** 1 International Vaccine Access Center, Department of International Health, Johns Hopkins Bloomberg School of Public Health, Baltimore, Maryland, United States of America; 2 Kenya Medical Research Institute/Centers for Disease Control and Prevention Public Health Collaboration, Nairobi and Kisumu, Kenya; 3 Influenza Division, Centers for Disease Control and Prevention, Atlanta, Georgia, United States of America; 4 Division of Global Disease Detection and Emergency Response, Center for Global Health, Centers for Disease Control and Prevention, Atlanta, Georgia, United States of America; 5 Centers for Disease Control and Prevention Regional Office for Central America and Panama, Guatemala City, Guatemala; 6 Influenza Program, Centers for Disease Control and Prevention Regional Office for Central America, Guatemala City, Guatemala; 7 Influenza Division, Centers for Disease Control and Prevention, Atlanta, Georgia, United States of America; 8 Center for Health Studies, Universidad del Valle de Guatemala, Guatemala City, Guatemala; 9 Kenya Medical Research Institute, Centre for Geographic Medicine Research–Coast, Kilifi, Kenya; 10 School of Life Sciences, University of Warwick, Coventry, United Kingdom; 11 Division of Preparedness and Emerging Infections, Centers for Disease Control and Prevention, Atlanta, Georgia, United States of America; University of Hong Kong, Hong Kong

## Abstract

**Background:**

Knowing the national disease burden of severe influenza in low-income countries can inform policy decisions around influenza treatment and prevention. We present a novel methodology using locally generated data for estimating this burden.

**Methods and Findings:**

This method begins with calculating the hospitalized severe acute respiratory illness (SARI) incidence for children <5 years old and persons ≥5 years old from population-based surveillance in one province. This base rate of SARI is then adjusted for each province based on the prevalence of risk factors and healthcare-seeking behavior. The percentage of SARI with influenza virus detected is determined from provincial-level sentinel surveillance and applied to the adjusted provincial rates of hospitalized SARI. Healthcare-seeking data from healthcare utilization surveys is used to estimate non-hospitalized influenza-associated SARI. Rates of hospitalized and non-hospitalized influenza-associated SARI are applied to census data to calculate the national number of cases. The method was field-tested in Kenya, and validated in Guatemala, using data from August 2009–July 2011. In Kenya (2009 population 38.6 million persons), the annual number of hospitalized influenza-associated SARI cases ranged from 17,129–27,659 for children <5 years old (2.9–4.7 per 1,000 persons) and 6,882–7,836 for persons ≥5 years old (0.21–0.24 per 1,000 persons), depending on year and base rate used. In Guatemala (2011 population 14.7 million persons), the annual number of hospitalized cases of influenza-associated pneumonia ranged from 1,065–2,259 (0.5–1.0 per 1,000 persons) among children <5 years old and 779–2,252 cases (0.1–0.2 per 1,000 persons) for persons ≥5 years old, depending on year and base rate used. In both countries, the number of non-hospitalized influenza-associated cases was several-fold higher than the hospitalized cases.

**Conclusions:**

Influenza virus was associated with a substantial amount of severe disease in Kenya and Guatemala. This method can be performed in most low and lower-middle income countries.

## Introduction

Influenza disease burden data are sparse in low and lower-middle income countries [1],[2],[3],[4],[5],[6]. Local data on influenza disease incidence and case counts are useful for decision makers in these countries to assess the public health importance of influenza, to identify high risk groups and regions, to allocate resources efficiently, and to consider the cost-effectiveness of preventive strategies, such as vaccination.

Determining the amount of influenza-associated severe disease will likely have greater public health significance in low and lower-middle income countries where mortality from infectious diseases is still high. Estimates of influenza-associated severe acute respiratory illness (SARI) incidence have been calculated in some low and lower-middle income countries from population-based surveillance [1],[3],[4],[7],[8],[9],[10]. These estimates are usually limited to the area where the surveillance is performed. However, in many countries there is regional variability in risk factors, epidemiology, and healthcare-seeking practices that can affect hospitalized influenza-associated SARI rates and make it challenging to extrapolate rates from a small area to the whole country. The more country-specific data that are utilized to estimate the total number of influenza cases, the more credible this estimate is likely to be to public health decision makers within the country.

We present here a novel, replicable methodology using locally-generated data for estimating the national number of cases of influenza-associated SARI in low and lower-middle income countries and provide results of the initial field test in Kenya. The method was also validated using different case-definitions for influenza-associated pneumonia from Guatemala.

## Methods

### Study site

Siaya District Hospital (SDH) is located in Siaya District, Nyanza Province in rural western Kenya. HIV prevalence in Nyanza Province was 13.9% among adults in 2009 [11]. Population-based surveillance at SDH is embedded in a larger Health and Demographic Surveillance System (HDSS) [12]. For this analysis, a case of SARI in children <5 years was defined as a modification of the WHO definition of pneumonia as any child, residing in a defined catchment area, hospitalized with cough or difficulty breathing and any one of the following: tachypnea for age group, unable to drink or breastfeed, vomits everything, convulsions, lethargic or unconscious, nasal flaring, grunting, oxygen saturation <90%, chest indrawing, or stridor in a calm child [13],[14]. SARI for persons ≥5 years was defined as any hospitalized case with cough, difficulty breathing, or chest pain during the previous 14 days. The denominator used to calculate rates was from Karemo Division, the division located closest to SDH. No other inpatient facilities exist in Karemo.

Kilifi District Hospital (KDH) is located in Kilifi district, Coast province in eastern Kenya. In 2009 among adults, Coast province had an HIV prevalence of 4.2% [11]. A HDSS is in operation within the district run by the KEMRI-Wellcome Trust Research Programme, which allows accurate determination of age-specific denominators [15],[ 16]. For most residents of this study area, KDH is the nearest inpatient facility; however, there are three other inpatient hospitals within the district. KDH only had population-based data for children <5 years for August 2009–July 2010. The same case definition of SARI in children was used as in Siaya. The base rates calculated for Kilifi were calculated using administrative sub-locations within 5 km of KDH.

Sentinel hospital influenza surveillance in Kenya was established in 2007 and took place in all 8 provinces. Specimens were collected from hospitalized patients who met the SARI case definition, which for persons ≥5 years old was defined as a fever of ≥38°C with cough or shortness of breath or difficulty breathing and for children was defined as given above [13]. Influenza vaccine is rarely used in the public or private sector in Kenya, as in other sub-Saharan African countries [17].

### Overview of burden calculation

**Figure 1:**
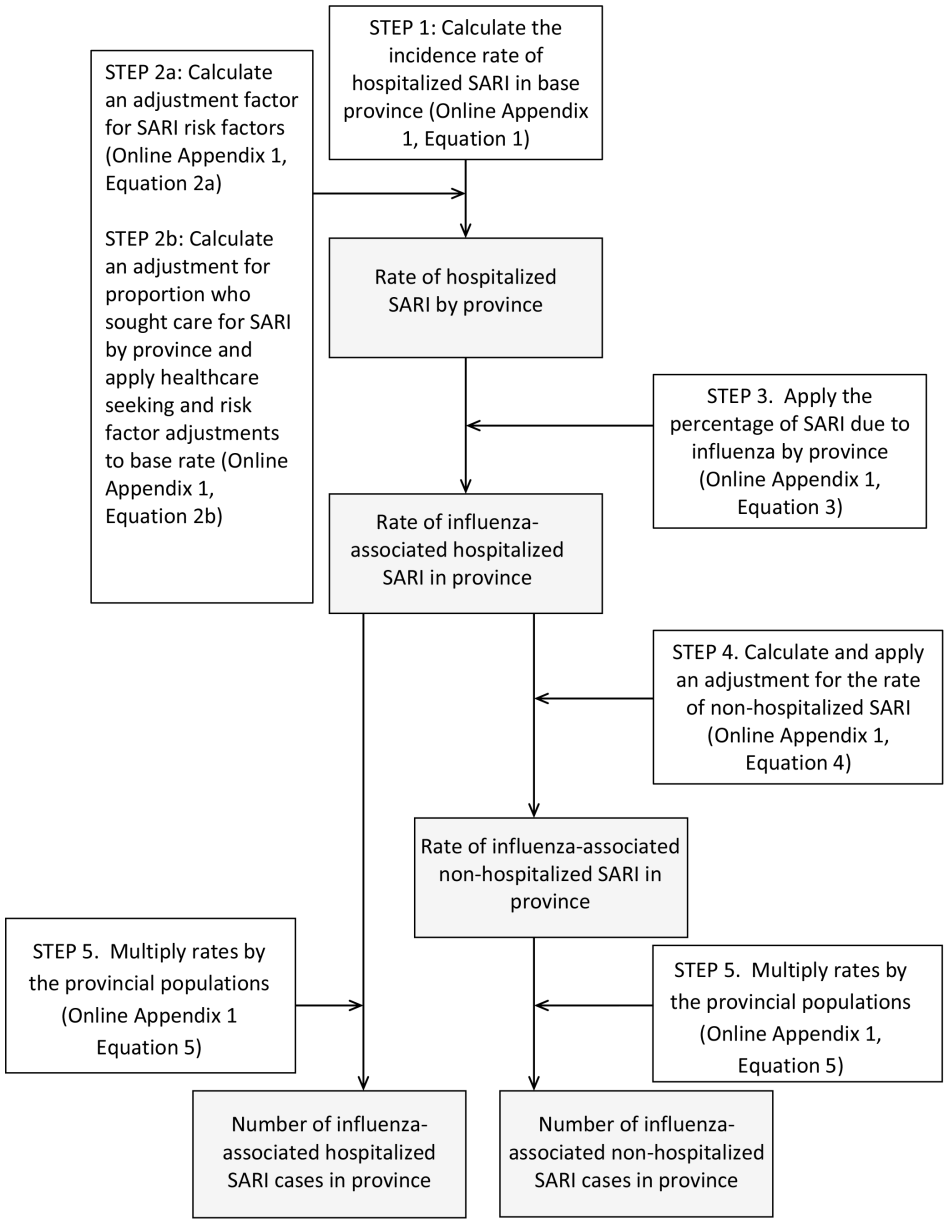
Overview of methodology for calculation of annual number of cases of influenza-associated Severe Acute Respiratory Illness (SARI) in a country. Data input steps are in white boxes and data output are in shaded boxes.

Figure 1 provides an overview of the methodology.

#### Step 1. Calculate incidence rate of hospitalized SARI from base province (Appendix S1, Equation 1)

From population-based surveillance sites with known catchment populations, we calculated the rate of hospitalized SARI, referred to as the ‘base rate.’ We used a catchment area of 5 kilometers from the district hospital as our denominator, because we felt persons within this area would be most likely to seek care at this facility and we could define the most accurate incidence, as healthcare utilization has been shown to decrease with greater distance from a health facility in Africa [18]. The number of SARI patients admitted to the surveillance hospital for each year of surveillance was divided by the age-specific hospital catchment populations.

#### Step 2a. Calculate an adjustment factor for SARI risk factors for each province (Appendix S1, Equation 2a)

We calculated an adjustment factor for each province in the country based on known risk factors for SARI adapted from a method used to calculate the global incidence of pneumonia in children by Rudan et al., which applied adjustments for five risk factors for pneumonia in low and lower-middle income countries, including malnutrition (weight-for-age z-score <−2), low birth weight (≤2500 g), non-exclusive breastfeeding (during the first 4 months of life), household air pollution (defined as using solid fuels), and crowding (defined as ≥5 people per household) [19]. Our adjustments were made based on the prevalence of these risk factors in each province and their relative risk for childhood pneumonia as defined by Rudan et al. based on their review of the literature (Table 1) [11],[ 20]. Because of the elevated risk for hospitalized SARI in HIV-infected persons, we added an adjustment for HIV prevalence using the same equation [21],[22],[23],[24],[25],[26]. We did not have data on HIV prevalence in children in Kenya, therefore we calculated this prevalence using an algorithm which took into account the prevalence of HIV-infected mothers from antenatal clinics and the expected vertical transmission rate, accounting for penetration of prevention-of-mother-to-child-transmission programs, and transmission through breastfeeding (Appendix S2). For persons ≥5 years old, the risk factors used for adjustment were household air pollution, crowding, and HIV prevalence. Due to a lack of studies found during a literature review of these risk factors for adult pneumonia, we assumed that the relative risk of SARI for each risk factor was the same as for children.

**Table 1:** Risk factors and associated relative risks of low and lower-middle income severe acute respiratory infection, and sources of data on risk factors by country.

Risk factor	Relative Risk (Reference Number)	Source of risk factor prevalence data Kenya	Source of risk factor prevalence data Guatemala
Malnutrition	1.8 (19)	Kenya DHS^1^ 2009	Guatemala ENSMI^2^ 2008–2009
Low birth weight	1.4 (19)	Kenya DHS^1^ 2009	Guatemala ENSMI^2^ 2008–2009
Non-exclusive breastfeeding	1.9 (19)	MICS UNICEF 2000	Guatemala ENSMI^2^ 2008–2009
Household air pollution^4^	1.8 (19)	Kenya DHS^1^ 2009	Guatemala ENSMI^2^ 2008–2009
Crowding	1.4 (19)	Kenya DHS^1^ 2003	Guatemala ENSMI^2^ 2008–2009
HIV Prevalence (Children <5 years)	7.2 (26)	Calculation in Appendix 2	N.A.
HIV Prevalence (Person ≥5 years)	5.64 (25)	Kenya DHS^1^ 2009	N.A.

1 Demographic and Health Survey.

2 Encuesta Nacional de Salud Materno-Infantil (National Survey of Maternal and Child Health.)

3 Multi-cluster indicator survey.

4 The prevalence of household air pollution was calculated by applying the proportion of persons cooking with solid fuels in rural and urban areas to the proportion of persons living in rural and urban areas for each province.

#### Step 2b. Apply adjustments for risk factors and for healthcare-seeking for SARI by province (Appendix S1, Equation 2b)

The rate of hospitalized SARI by province was calculated by applying the risk-factor adjustment described in Step 2a and further adjusting for healthcare-seeking practices using a ratio of healthcare-seeking in the base province to each other province. Since 2007, national Demographic and Health Surveys (DHS) have included a standardized question that asks about healthcare-seeking practices for acute respiratory illness (ARI) [11]. The question currently asks caretakers ‘if their children under age five had been ill in the two weeks preceding the survey with a cough accompanied by short, rapid breathing or difficulty breathing which the mother considered to be chest-related.’ If yes, the caretaker is asked if he or she ‘sought advice or treatment from a health facility or a provider’, which excludes pharmacies, shops and traditional healers. The percentage of children with ARI in the past two weeks who sought care at a health facility or provider is reported by administrative region in the country (e.g., province). As there was no question in the DHS asking about healthcare-seeking for adults and since the relative rather than absolute healthcare-seeking by province was more important for the adjustment, we used the same proportion for adults who sought care as for children from the DHS. We also assumed that healthcare-seeking for SARI in a province would be proportional to healthcare-seeking for ARI as defined in the DHS, and applied the ratio of these percentages to further adjust the rate of SARI for each province compared to the base rate province.

#### Step 3. Apply the percentage of SARI associated with influenza by province

(Appendix S1, Equation 3.) The percentage of hospitalized SARI associated with influenza A and B viruses was obtained from established sentinel influenza surveillance sites. In Kenya, each province had one sentinel surveillance site and in Guatemala only the two provinces with population-based SARI surveillance had influenza-specific data available for this analysis. In Kenya, for provinces with <25 cases of SARI during a surveillance year, we considered the estimate as unstable due to small numbers or insensitive surveillance, and so used a weighted average of the percentage of hospitalized influenza-associated SARI from provinces with ≥25 cases, weighted by the number of samples taken. We applied these percentages to the rate of hospitalized SARI by province to determine the rate of hospitalized influenza-associated SARI for each province.

#### Step 4. Calculate and apply an adjustment for the rate of non-hospitalized SARI

(Appendix S1, Equation 4.) Using data from published healthcare utilization surveys asking about healthcare-seeking practices for pneumonia, we estimated the rate of non-hospitalized SARI [27],[28],[29]. In these surveys, ‘pneumonia’ was defined as cough or difficulty breathing for more than two days or a diagnosis of ‘pneumonia’ by a healthcare worker. This estimation was based on the assumption that those patients have the same severity of illness, but did not seek care due to lack of access. We felt that compared to the DHS, the healthcare utilization survey healthcare-seeking questions were more relevant for SARI, as the questions focused on pneumonia, rather than the more nonspecific ARI question of the DHS; we expected more healthcare-seeking for more severe episodes like SARI than for all ARI. In addition, the healthcare utilization survey questions were asked for both children and adults. In both countries the healthcare utilization survey defined pneumonia as, ‘cough or difficulty breathing that lasted more than 2 days or a diagnosis of pneumonia given by a doctor or professional healthcare provider in the last year’. Respondents who reported a pneumonia episode in the past year were asked about healthcare-seeking. For this analysis, we considered the percentage of patients with pneumonia who sought care at a hospital as indicative of the percentage of persons who would have access to a hospital if they were to have an episode of influenza-associated SARI. Because healthcare utilization survey data was only available in one province in Kenya and two departments in Guatemala, we adjusted the healthcare utilization survey-derived percentage of those who sought care at a hospital for pneumonia to each province by applying the same ratio of healthcare-seeking for ARI from the DHS that we used in Step 2b; although in this case the ratio was for each province divided by the province in which the healthcare utilization survey was done (which may or may not have been the same as the province where the base rate of SARI was obtained).

#### Step 5. Multiply rates by the provincial populations (Appendix S1, Equation 5.)

The age-specific provincial populations were multiplied by the rates of hospitalized and non-hospitalized influenza-associated SARI to determine the number of cases for each province. The populations of the provinces were obtained from the most recent national census data, with projected annual population growth [30],[31]. The numbers of cases in each province were summed to calculate the national number of cases.

#### Uncertainty ranges

Confidence intervals were estimated by bootstrapping each data input in steps 1–4 above 1,000 times which resulted in 1,000 estimates of the number of hospitalized and non-hospitalized influenza-associated cases in the country. The upper and lower limits of the 95% confidence intervals were the 2.5th and 97.5th percentiles of these estimates, respectively. Confidence intervals are not symmetric because some inputs were re-sampled on the margins of the parameter space (e.g. proportions close to 0 or 1), reducing their potential variability in only one direction.

#### Guatemala

We validated this methodology in Guatemala using the slightly different case definitions used in surveillance there. The Centers for Disease Control and Prevention (CDC), in conjunction with the University of the Valley of Guatemala (UVG) and the Guatemalan Ministry of Public Health and Social Assistance, conducted population-based surveillance for pneumonia in two departments --in Santa Rosa, a rural lowland department in southeast Guatemala, at the National Hospital of Cuilapa, and in Quetzaltenango, a semi-urban highland department in western Guatemala, at Western National Hospital. During the 2009 influenza pandemic year, immunization for pH1N1 was reasonably high, but coverage for seasonal strains is typically low [32]. At both Guatemalan sites, a case of pneumonia was defined as a hospitalized patient, residing in the hospital's pre-defined catchment area with at least one sign of an acute infection (e.g. fever, abnormal white blood cell count, hypothermia) and at least one sign or symptom of a respiratory tract illness (e.g. cough, rapid breathing, production of phlegm, chest pain, difficulty breathing) [33]. All patients meeting the pneumonia case definition were tested for influenza virus using real-time PCR [34]. Besides Quetzaltenango and Santa Rosa, no other sites had complete influenza sentinel surveillance data available. In Guatemala, there were a few deviations from the methodology described above. In Step 1, the base rate included an adjustment for the proportion of SARI cases hospitalized at non-surveillance hospitals within the catchment area. Also, there was no recent DHS data for Guatemala, but a similar survey (National Survey of Maternal and Child Health [ENSMI]) contained data on healthcare-seeking practices (Step 2b) and risk factor prevalence (Step 2a) [35]. Finally, because prevalence of HIV remains low (<1%), no adjustments were made for HIV in Step 2a.

### Ethical Review

Approval of protocols and consent forms for ongoing surveillance, where relevant, were obtained by the respective ethical review committees of CDC, KEMRI and Wellcome-Trust, and UVG.

## Results

### Kenya

In the first year, the base rates of hospitalized SARI for children <5 years old in Siaya and Kilifi were 55.6 and 27.3 per 1,000 persons, respectively, and for persons ≥5 years old were 3.1 per 1,000 persons in Siaya (Table 2 and Table 3). In the second year, rates were slightly lower in children and slightly higher in persons ≥5 years. Risk factors for SARI and healthcare-seeking practices varied by province. This led to variability in the rates of hospitalized SARI by province. For example, during the first year (Karemo base rate), the point estimates of hospitalized SARI rates by province ranged from 16.6–55.6 per 1,000 persons for children and 1.4–3.1 per 1,000 for persons ≥5 years old.

**Table 2:** Rate (per 1,000) and 95% confidence limits of influenza-associated severe acute respiratory illness (SARI) among children <5 years of age in Kenya, August 2009 to July 2011, using the Karemo division denominator (see methods).

			August 2009–July 2010^3^		August 2010–July 2011^4^	
Province	Adjustment for Risk Factor prevalence and DHS Healthcare-seeking for ARI compared with base-rate province^1^	Percent of pneumonia cases hospitalized from HUS^2^	Hospitalized rate per 1,000 (95% CL)	Non-hospitalized rate per 1,000 (95% CL)	Hospitalized rate per 1,000 (95% CL)	Non-hospitalized rate per 1,000 (95% CL)
Central	0.72	0.40	5.4 (2.8–8.5)	8.1 (6.1–12.2)	3.2 (1.7–5.3)	4.8 (3.6–6.8)
Coast	0.78	0.50	4.6 (2.5–7.3)	4.7 (2.8–7.6)	3.0 (1.9–4.4)	3.0 (2.0–4.4)
Eastern	0.85	0.46	1.7 (0.0–5.8)	2.0 (0.0–7.3)	2.3 (0.9–4.1)	2.7 (1.1–4.9)
Nairobi	0.30	0.60	0.6 (0.2–1.2)	0.4 (0.1–0.9)	1.1 (0.5–1.8)	0.7 (0.4–1.3)
North Eastern	0.95	0.54	5.7 (2.2–12.6)	4.9 (2.3–8.2)	2.2 (0.9–5.0)	1.9 (1.0–2.9)
**Nyanza**	**1.00**	**0.48**	**3.9 (3.1–4.7)**	**4.2 (2.7–7.3)**	**3.0 (2.2–3.7)**	**3.2 (2.1–5.5)**
Rift Valley	0.86	0.51	7.7 (5.2–11.0)	7.4 (5.0–12.8)	4.2 (2.9–6.1)	4.1 (2.5–6.5)
Western	0.56	0.40	3.6 (2.2–5.7)	5.4 (3.8–8.0)	2.5 (1.4–4.0)	3.7 (2.6–5.7)

DHS is Demographic and Health Survey; ARI is acute respiratory illness; HUS is Healthcare Utilization Survey; CL is confidence limit.

The base province (Nyanza), where Karemo division is located, is bolded.

1 This adjustment factor is based on 6 risk factors for SARI and healthcare-seeking behaviors, adjusting the rate of the base province in bold to the other provinces. (
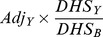
 from Equation 2a).

2 This adjustment factor is used to estimate the rate of non-hospitalized cases assumed to be of the same severity as hospitalized cases. (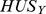 from Equation 4).

3 Base rate for Karemo division surveillance (Nyanza province) among children <5 in August 2009 to July 2010 is 55.55 per 1,000 persons per year.

4 Base rate for Karemo division surveillance (Nyanza province) among children <5 in August 2010 to July 2011 is 44.63 per 1,000 persons per year.

**Table 3:** Rate (per 1,000) and 95% confidence limits of influenza-associated severe acute respiratory illness (SARI) among persons ≥5 years of age in Kenya, August 2009 to July 2011, using the Karemo division denominator (see methods).

			August 2009–July 2010^3^		August 2010–July 2011^4^	
Province	Adjustment for Risk Factor prevalence and DHS Healthcare-seeking for ARI compared with base-rate province^1^	Percent of pneumonia cases hospitalized from HUS^2^	Hospitalized rate per 1,000 (95% CL)	Non-hospitalized rate per 1,000 (95% CL)	Hospitalized rate per 1,000 (95% CL)	Non-hospitalized rate per 1,000 (95% CL)
Central	0.43	0.28	0.2 (0.0–0.4)	0.5 (0.1–1.1)	0.2 (0.1–0.3)	0.4 (0.1–0.8)
Coast	0.51	0.35	0.2 (0.1–0.3)	0.3 (0.2–0.6)	0.2 (0.0–0.4)	0.3 (0.1–0.8)
Eastern	0.51	0.33	0.2 (0.1–0.3)	0.3 (0.2–0.6)	0.2 (0.1–0.3)	0.4 (0.3–0.7)
Nairobi	0.53	0.43	0.2 (0.1–0.3)	0.2 (0.1–0.4)	0.2 (0.2–0.3)	0.3 (0.2–0.5)
North Eastern	0.68	0.38	0.2 (0.1–0.4)	0.4 (0.2–0.6)	0.3 (0.1–0.5)	0.4 (0.3–0.7)
**Nyanza**	**1.00**	**0.34**	**0.3 (0.2–0.4)**	**0.6 (0.3–1.2)**	**0.4 (0.3–0.5)**	**0.8 (0.5–1.6)**
Rift Valley	0.67	0.36	0.2 (0.1–0.3)	0.4 (0.2–0.7)	0.2 (0.0–0.5)	0.4 (0.0–1.1)
Western	0.56	0.28	0.2 (0.1–0.4)	0.6 (0.2–1.1)	0.2 (0.1–0.4)	0.6 (0.3–1.1)

DHS is Demographic and Health Survey; ARI is acute respiratory illness; HUS is Healthcare Utilization Survey; CL is confidence limit.

The base province (Nyanza), where Karemo division is located, is bolded.

1 This adjustment factor is based on 3 risk factors for SARI and healthcare-seeking behaviors, adjusting the rate of the base province in bold to the other provinces. (
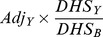
 from Equation 2a).

2 This adjustment factor is used to estimate the rate of non-hospitalized cases assumed to be of the same severity as hospitalized cases. (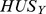 from Equation 4).

3 Base rate for Karemo division surveillance (Nyanza province) among persons ≥5 in August 2009 to July 2010 is 3.13 per 1,000 persons per year.

4 Base rate for Karemo division surveillance (Nyanza province) among persons ≥5 in August 2010 to July 2011 is 3.25 per 1,000 persons per year.

**Figure 2:**
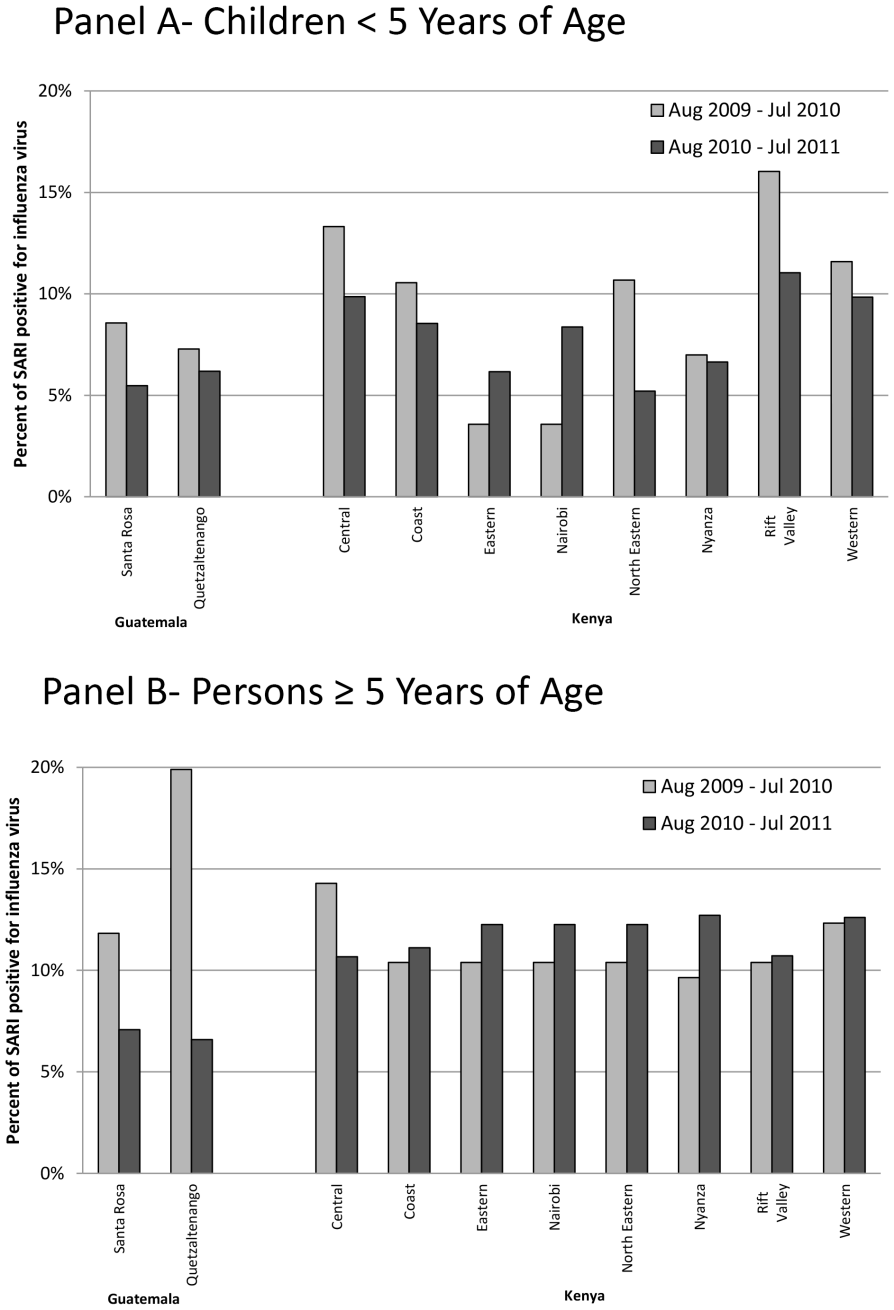
Percentage of severe acute respiratory infections (SARI) that tested positive for influenza by Guatemalan department and Kenyan province. August 2009-July 2011. Panel A-Children <5 years old. Panel B- Persons ≥5 years old.

The percentage of SARI with influenza virus detected varied by year and province (Figure 2). In Kenyan children, this percentage ranged from 3.6% to 16.0%. In Kenyan persons ≥5 years, the percentage ranged from 9.6% to 14.3%. The rates of influenza-associated hospitalized SARI were higher among children <5 years old than among persons ≥5 years old in both sites. Rates varied by province (Table 2 and Table 3). Forty-eight percent of children and 34% of older persons who reported pneumonia sought care at any hospital [27]. After adjustment, the rate of non-hospitalized influenza-associated SARI was higher than hospitalized influenza-associated SARI for persons ≥5 years old in Kenya in all provinces (Table 3), and for children in Kenya in most provinces (Table 2).

In Kenya (2009 population of 38.6 million persons), the point estimates for the annual number of hospitalized influenza-associated SARI cases ranged from 17,129–27,659 for children <5 years old (2.9–4.7 per 1,000 persons) and 6,882–7,836 for persons ≥5 years old (0.21–0.24 per 1,000 persons), depending on year and base rate used (Table 4). The point estimates for the annual number of non-hospitalized influenza-associated SARI cases ranged from 19,798–30,275 (3.3–5.1 per 1,000 persons) for children <5 years old and 13,592–15,270 for persons ≥5 years old (0.42–0.47 per 1,000 persons), depending on year and base rate used (Table 4). The uncertainty ranges around the annual burden estimates are shown in Table 4.

**Table 4:** National average annual rate (per 1,000 persons) and number of cases (and 95% Confidence Limits) of Influenza-Associated SARI in Kenya, August 2009 to July 2011.

	<5 years old^1^		≥5 years old^2^		All persons	
Base Province	Hospitalized	Non-hospitalized	Hospitalized	Non-hospitalized	Hospitalized	Non-hospitalized
August 2009–July 2010						
Rate per 1,000 (95% confidence limit)						
Siaya	4.7 (3.5–6.2)	5.1 (3.5–8.1)	0.2 (0.2–0.3)	0.4 (0.2–0.8)	1.1 (0.9–1.6)	1.4 (0.9–2.4)
Kilifi	2.9 (2.1–4.0)	3.3 (2.3–5.4)	N/A	N/A	N/A	N/A
Annual Number of Cases (95% confidence limit)						
Siaya*	27,659 (20,774–36,583)	30,275 (21,543–49,065)	6,882 (4,893–9,598)	13,592 (8,611–27,035)	34,541 (25,667–46,181)	43,867 (30,154–76,100)
Kilifi*	17,129 (12,645–23,672)	19,918 (14,031–31,659)	N/A	N/A	N/A	N/A
August 2010–July 2011						
Rate per 1,000 (95% confidence limit)						
Siaya	3.0 (2.3–3.9)	3.3 (2.4–5.2)	0.2 (0.2–0.4)	0.5 (0.3–0.9)	0.7 (0.5–0.9)	0.9 (0.6–1.6)
Kilifi	N/A	N/A	N/A	N/A	N/A	N/A
Annual Number of Cases (95% confidence limit)						
Siaya^3^	17,926 (14,006–23,795)	19,798 (13,706–31,117)	7,836 (5,330–11,402)	15,270 (8,818–28,897)	25,762 (19,336–35,197)	35,068 (22,524–60,014)
Kilifi^3^	N/A	N/A	N/A	N/A	N/A	N/A

1 Kenya under-5 population in 2009: 5,939,306.

2 Kenya 5 years and older population in 2009: 32,649,705.

3 Using the denominators of the smaller populations closer to the hospital. Note that Kilifi only provided data for August 2009–July 2010 for children so that all other periods are indicated as N/A, not applicable.

### Guatemala

In Guatemala in the first year, the base rates of hospitalized pneumonia for children <5 years old in Quetzaltenango and Santa Rosa were 9.7 and 8.7 per 1,000 persons, respectively, and for persons ≥5 years old were 0.67 and 0.86 per 1,000 persons, respectively (Table S1, Table S2, Table S3, Table S4). In the second year, rates were slightly higher for children and similar for older persons. Risk factors and healthcare-seeking practices varied by province. This led to variability in rates of hospitalized pneumonia by department. For example, using the Santa Rosa base rate in the first year, hospitalized pneumonia rates by department ranged from 8.8–15.7 per 1,000 persons for children <5 years old and 0.7–1.5 per 1,000 for persons ≥5 years old.

The percentage of pneumonia cases with influenza virus detected varied by year and department (Figure 2). In Guatemalan children, this percentage ranged from 5.5% to 8.6%. In persons ≥5 years, this percentage ranged from 6.6% to 19.9%. In the Santa Rosa area, 28% of children and 11% of older persons who reported pneumonia sought care at any hospital [28]. At the Quetzaltenango site, 40% of children and 16% of older persons who reported pneumonia sought care at any hospital (Oliver Morgan, personal communication).

In Guatemala (2011 population of 14.7 million persons), the point estimates for the annual number of hospitalized cases of influenza-associated pneumonia ranged from 1,065–2,259 (0.5–1.0 per 1,000 persons) among children <5 years old and 779–2,252 cases (0.1–0.2 per 1,000 persons) for persons ≥ 5 years old, depending on year and base rate used. The point estimates for the annual number of non-hospitalized cases of influenza-associated pneumonia ranged from 2,033–4,312 (0.9–2.0 per 1,000 persons) among children <5 years old and 4,592–13,268 cases for persons ≥5 years old (0.4–1.1 per 1,000 persons), depending on year and base rate used.

## Discussion

Our method for calculating the national number of influenza-associated SARI has several advantages. First, it incorporates as much data from within the country as available, adjusting for regional differences in SARI risk factors and influenza prevalence. In order to make informed policy decisions, countries likely perceive data generated within their own country as more relevant and persuasive [36]. For countries without population-based surveillance, however, base rates of SARI can be used from epidemiologically-similar neighboring countries. Second, our approach emphasizes the use of sentinel laboratory-based surveillance data and reinforces the need to assure high-quality influenza surveillance [13]. Third, the estimate incorporates both hospitalized and non-hospitalized cases. Estimates of hospitalized cases alone can be utilized for planning care and treatment costs. However, in most low and lower-middle income countries, healthcare-seeking practices at hospitals can be low, particularly in rural areas where many persons die at home [12],[27],[29]. The full burden of severe influenza disease in low and lower-middle income countries, and the potential impact of interventions like vaccination, requires quantification of non-hospitalized cases as well.

For children <5 years of age, we found incidences of influenza-associated SARI similar in magnitude to those presented for low and lower-middle income countries from the same region in a recent systematic review of global influenza burden in children 3]. For persons ≥5 years old, the rates we found for influenza-associated SARI are similar to estimates from Bangladesh, Thailand, and another part of western Kenya [1],[7],[10]. Of note, our estimates included the period when pandemic H1N1 appeared in both countries, which might have led to changes in healthcare-seeking practices and influenza incidence that were not representative of normal years [33],[37]. Kenya was unique in having two different sites performing laboratory-based, population-based surveillance for hospitalized influenza in children, which allowed us to further check the accuracy of our methodology [1],[38]. When we used surveillance in Kilifi in Coast Province for the base rate in our method, we estimated a rate of hospitalized influenza-associated SARI of 2.3 per 1,000 (95% CI: 1.6–3.4) in Nyanza Province (Table S5), which is similar, with overlapping confidence intervals, to that found from a published report in Nyanza Province (1.4 per 1,000, 95% CI 1.2–1.7); the differences could be due to slightly different case definitions, catchment designs, and years of inclusion [1].

Alternate methods have been used to estimate national influenza case counts. One methodology is to use administrative data, such as national hospital discharge summaries based on ICD coding or vital statistics registries, to ascertain the number of hospitalizations and deaths associated with respiratory diseases. Such models have been used in the U.S., Canada, Hong Kong and South Africa [8],[39],[40],[41],[42],[43]. However, reliable longitudinal administrative data is not available in most low and lower-middle income countries. A second methodology is to perform healthcare utilization surveys at each sentinel surveillance site to define the incidence of both hospitalized and non-hospitalized SARI, since healthcare-seeking practices can differ regionally. This approach has been used in Bangladesh and Thailand [7],[9],[10]. While this approach can succeed in a research-oriented, well-resourced setting, this is not readily applicable to most low and lower-middle income countries with recently established sentinel influenza surveillance. Properly done healthcare utilization surveys are both time- and resource-intensive [27],[28],[29]. A more economical and perhaps more accurate approach would be to use data from one well-executed healthcare utilization survey, and adjust the results for every province using readily available data (e.g. DHS surveys), as done in our method [11].

Our method has several potential limitations. First, the estimates are sensitive to the base rate of SARI used. For example, to calculate incidence fully it is important to capture all the cases within a defined catchment area, which might mean enrolling children from multiple hospitals that serve the catchment area. Also, because healthcare utilization is suboptimal in many low income countries, rates tend to be higher, and perhaps more accurate of the true incidence, when using catchment populations closer to the facilities where cases are captured [18],[38],[44],[45]. We chose to use catchment areas within a 5 kilometers radius from our base rate surveillance hospitals in Kenya. Second, in Kenya, different case definitions were used for SARI among adults in the population-based surveillance and sentinel influenza surveillance, the main difference being that the latter required documented fever [13]. It has been shown that the adult SARI case definition commonly used for sentinel surveillance misses many cases of influenza-associated hospitalization, and we considered it important for a burden estimate to use a broader, more sensitive case definition for the base rate [46]. Even so, we likely missed some non-respiratory influenza-associated hospitalizations in which influenza might have exacerbated underlying chronic illnesses [47]. Third, we assumed that the severity of non-hospitalized SARI was equivalent to hospitalized SARI, which might not have been the case. In addition, because countries may use different case definitions for SARI, care should be taken when comparing incidence estimated by this tool between countries. For example, we used Guatemalan data to validate our method, despite their use of a pneumonia case definition that differed from the SARI case definitions used in Kenya. While this prevents direct comparison of influenza case counts and incidence between Guatemala and Kenya, or other countries with SARI surveillance, the data are internally consistent for each country and demonstrate flexibility of the methodology in being able to use locally available surveillance definitions. Lastly, our methodology did not produce estimates of influenza-associated mortality, which are useful to decision makers.

Country-level influenza disease estimates will become increasingly important to Ministries of Health over the coming years. As the role of influenza as a major public health concern comes into sharper focus, countries will need representative and ideally country-specific data to make decisions about treatment and preventive strategies, including targeted vaccination with currently available influenza vaccines. Knowing the number of severe influenza cases in their countries, as well as the associated costs of illness, will afford decision makers the type of data needed to formulate sound national policies.

## Supporting Information

Table S1 **Rates (per 1,000) of hospitalized and non-hospitalized influenza-associated pneumonia in Guatemala among children <5 years of age, August 2009 to July 2011.** Santa Rosa (bolded) surveillance was used for the base rate as well as the healthcare utilization survey.
(DOCX)

Table S2 **Rates (per 1,000) of hospitalized and non-hospitalized influenza-associated pneumonia in Guatemala among persons ≥5 years of age, August 2009 to July 2011.** Santa Rosa (bolded) surveillance was used for the base rate as well as the healthcare utilization survey.
(DOCX)

Table S3 **Rates (per 1,000) of hospitalized and non-hospitalized influenza-associated pneumonia in Guatemala among children <5 years of age, August 2009 to July 2011.** Quetzaltenango (bolded) surveillance was used for the base rate as well as the healthcare utilization survey.
(DOCX)

Table S4 **Rates (per 1,000) of hospitalized and non-hospitalized influenza-associated pneumonia in Guatemala among persons ≥5 years of age, August 2009 to July 2011.** Quetzaltenango (bolded) surveillance was used for the base rate as well as the healthcare utilization survey.
(DOCX)

Table S5 **Rates and 95% confidence limits (per 1,000) of hospitalized and non-hospitalized influenza-associated severe acute respiratory illness (SARI) in Kenyan children <5 years of age, August 2009 to July 2010.** Kilifi, Coast province (bolded) surveillance was used for the base rate.
(DOCX)

Appendix S1 **Equations used in calculation of annual number of cases of influenza-associated severe acute respiratory infection (SARI) cases.**
(DOCX)

Appendix S2 **Equation used to calculate HIV prevalence in children.** PMTCT is Prevention of Mother to Child Transmission.
(DOCX)
