# Supplementary material for: Estimation of the National Disease Burden of Influenza-Associated Severe Acute Respiratory Illness in Kenya and Guatemala: A Novel Methodology
Source: PLoS One. 2013 Feb 27;8(2):e56882. doi: 10.1371/journal.pone.0056882 (PMC3584100; doi:10.1371/journal.pone.0056882)
Supplement: Table S1 — Rates (per 1,000) of hospitalized and non-hospitalized influenza-associated pneumonia in Guatemala among children <5 years of age, August 2009 to July 2011. Santa Rosa (bolded) surveillance was used for the base rate as well as the healthcare utilization survey. (DOCX) [file pone.0056882.s001.docx]

**Table S1**- Rates (per 1,000) of hospitalized and non-hospitalized influenza-associated pneumonia in Guatemala among children < 5 years of age, August 2009 to July 2011. Santa Rosa (bolded) surveillance was used for the base rate as well as the healthcare utilization survey.

| **Department** | **Adjustment for Risk Factor prevalence and DHS Healthcare-seeking for ARI compared with base-rate province^1^** | **Percent of pneumonia cases hospitalized from HUS^2^** | **Hospitalized Rate (per 1,000) Aug 2009-July 2010^3^** | **Non-Hospitalized Rate (per 1,000) Aug 2009-July 2010^3^** | **Hospitalized Rate (per 1,000) Aug 2010-July 2011^4^** | **Non-Hospitalized Rate (per 1,000) Aug 2010-July 2011^4^** |
| --- | --- | --- | --- | --- | --- | --- |
| Guatemala | 0.98 | 0.37 | 0.81 | 1.40 | 0.38 | 0.66 |
| El Progreso | 0.91 | 0.31 | 0.75 | 1.71 | 0.35 | 0.81 |
| Sacatepequez | 1.24 | 0.37 | 1.02 | 1.71 | 0.48 | 0.81 |
| Chimaltenango | 1.38 | 0.34 | 1.14 | 2.21 | 0.54 | 1.04 |
| Escuintla | 1.09 | 0.34 | 0.90 | 1.75 | 0.42 | 0.82 |
| Solola | 1.42 | 0.33 | 1.17 | 2.36 | 0.55 | 1.11 |
| Totonicapan | 1.59 | 0.37 | 1.31 | 2.23 | 0.62 | 1.05 |
| Suchitepequez | 1.04 | 0.32 | 0.86 | 1.80 | 0.40 | 0.85 |
| Retalhuleu | 1.17 | 0.37 | 0.97 | 1.62 | 0.46 | 0.76 |
| San Marcos | 1.04 | 0.28 | 0.86 | 2.24 | 0.41 | 1.06 |
| Huehuetenango | 1.48 | 0.35 | 1.22 | 2.24 | 0.58 | 1.06 |
| Quiche | 1.63 | 0.35 | 1.34 | 2.48 | 0.63 | 1.17 |
| Baja Verapaz | 1.55 | 0.45 | 1.28 | 1.59 | 0.60 | 0.75 |
| Alta Verapaz | 1.22 | 0.33 | 1.01 | 2.02 | 0.48 | 0.95 |
| Peten | 1.31 | 0.36 | 1.08 | 1.91 | 0.51 | 0.90 |
| Izabal | 1.11 | 0.30 | 0.92 | 2.11 | 0.43 | 1.00 |
| Zacapa | 1.13 | 0.31 | 0.93 | 2.07 | 0.44 | 0.97 |
| Chiquimula | 1.47 | 0.35 | 1.21 | 2.25 | 0.57 | 1.06 |
| Jalapa | 1.43 | 0.34 | 1.18 | 2.31 | 0.56 | 1.09 |
| Jutiapa | 1.22 | 0.32 | 1.01 | 2.18 | 0.47 | 1.03 |
| Quetzaltenango | 1.16 | 0.39 | 0.96 | 1.51 | 0.45 | 0.71 |
| **Santa Rosa** | **1.00** | **0.28** | **0.83** | **2.09** | **0.39** | **0.98** |

1 This adjustment factor is based on 5 risk factors for ALRI and healthcare-seeking behaviors, adjusting the rate of the base province in bold to the other provinces. (${Adj}_{Y}$ from Equation 2a). Data available from National Survey of Maternal and Child Health 2008-2009 (Encuesta Nacional de Salud Materno-Infantil [ENSMI] 2008-2009). ARI is acute respiratory illness.

2 This adjustment factor is used to estimate the rate of non-hospitalized cases assumed to be of the same severity as hospitalized cases. HUS is Healthcare Utilization Survey. (${HUS}_{Y}$ from Equation 4).

3 Santa Rosa base rate for children < 5 years in August 2009 to July 2010 is 9.65 per 1,000

4 Santa Rosa base rate for children < 5 years in August 2010 to July 2011 is 7.11 per 1,000
