# Supplementary material for: Estimation of the National Disease Burden of Influenza-Associated Severe Acute Respiratory Illness in Kenya and Guatemala: A Novel Methodology
Source: PLoS One. 2013 Feb 27;8(2):e56882. doi: 10.1371/journal.pone.0056882 (PMC3584100; doi:10.1371/journal.pone.0056882)
Supplement: Table S2 — Rates (per 1,000) of hospitalized and non-hospitalized influenza-associated pneumonia in Guatemala among persons ≥5 years of age, August 2009 to July 2011. Santa Rosa (bolded) surveillance was used for the base rate as well as the healthcare utilization survey. (DOCX) [file pone.0056882.s002.docx]

**Table S2**- Rates (per 1,000) of hospitalized and non-hospitalized influenza-associated pneumonia in Guatemala among persons ≥ 5 years of age, August 2009 to July 2011. Santa Rosa (bolded) surveillance was used for the base rate as well as the healthcare utilization survey.

| **Department** | **Adjustment for Risk Factor prevalence and DHS Healthcare-seeking for ARI compared with base-rate province^1^** | **Percent of pneumonia cases hospitalized from HUS^2^** | **Hospitalized Rate (per 1,000) Aug 2009-July 2010^3^** | **Non-Hospitalized Rate (per 1,000) Aug 2009-July 2010^3^** | **Hospitalized Rate (per 1,000) Aug 2010-July 2011^4^** | **Non-Hospitalized Rate (per 1,000) Aug 2010-July 2011^4^** |
| --- | --- | --- | --- | --- | --- | --- |
| Guatemala | 0.87 | 0.14 | 0.08 | 0.50 | 0.05 | 0.34 |
| El Progreso | 0.91 | 0.11 | 0.08 | 0.65 | 0.06 | 0.44 |
| Sacatepequez | 1.19 | 0.14 | 0.11 | 0.67 | 0.07 | 0.45 |
| Chimaltenango | 1.31 | 0.13 | 0.12 | 0.82 | 0.08 | 0.55 |
| Escuintla | 1.05 | 0.13 | 0.10 | 0.66 | 0.07 | 0.45 |
| Solola | 1.55 | 0.12 | 0.14 | 1.00 | 0.10 | 0.67 |
| Totonicapan | 1.66 | 0.14 | 0.15 | 0.95 | 0.10 | 0.64 |
| Suchitepequez | 1.23 | 0.12 | 0.11 | 0.82 | 0.08 | 0.55 |
| Retalhuleu | 1.40 | 0.14 | 0.13 | 0.79 | 0.09 | 0.53 |
| San Marcos | 1.18 | 0.10 | 0.11 | 0.94 | 0.07 | 0.63 |
| Huehuetenango | 1.60 | 0.13 | 0.15 | 0.96 | 0.10 | 0.65 |
| Quiche | 1.73 | 0.13 | 0.16 | 1.05 | 0.11 | 0.70 |
| Baja Verapaz | 1.93 | 0.17 | 0.18 | 0.88 | 0.12 | 0.59 |
| Alta Verapaz | 1.58 | 0.12 | 0.15 | 1.02 | 0.10 | 0.69 |
| Peten | 1.50 | 0.14 | 0.14 | 0.88 | 0.09 | 0.59 |
| Izabal | 1.10 | 0.11 | 0.10 | 0.78 | 0.07 | 0.53 |
| Zacapa | 1.07 | 0.12 | 0.10 | 0.75 | 0.07 | 0.50 |
| Chiquimula | 1.37 | 0.13 | 0.13 | 0.83 | 0.09 | 0.56 |
| Jalapa | 1.35 | 0.13 | 0.12 | 0.86 | 0.08 | 0.58 |
| Jutiapa | 1.18 | 0.12 | 0.11 | 0.81 | 0.07 | 0.54 |
| Quetzaltenango | 1.38 | 0.15 | 0.13 | 0.74 | 0.09 | 0.50 |
| **Santa Rosa** | **1.00** | **0.11** | **0.09** | **0.77** | **0.06** | **0.52** |

1 This adjustment factor is based on 2 risk factors for ALRI and healthcare-seeking behaviors, adjusting the rate of the base province in bold to the other provinces. (${Adj}_{Y}$ from Equation 2a). Data available from National Survey of Maternal and Child Health 2008-2009 (Encuesta Nacional de Salud Materno-Infantil [ENSMI] 2008-2009). ARI is acute respiratory illness.

2 This adjustment factor is used to estimate the rate of non-hospitalized cases assumed to be of the same severity as hospitalized cases. HUS is Healthcare Utilization Survey. (${HUS}_{Y}$ from Equation 4).

3 Santa Rosa base rate for older participants in August 2009 to July 2010 is 0.67 per 1,000

4 Santa Rosa base rate for older participants in August 2010 to July 2011 is 0.75 per 1,000
