# Supplementary material for: Estimation of the National Disease Burden of Influenza-Associated Severe Acute Respiratory Illness in Kenya and Guatemala: A Novel Methodology
Source: PLoS One. 2013 Feb 27;8(2):e56882. doi: 10.1371/journal.pone.0056882 (PMC3584100; doi:10.1371/journal.pone.0056882)
Supplement: Table S3 — Rates (per 1,000) of hospitalized and non-hospitalized influenza-associated pneumonia in Guatemala among children <5 years of age, August 2009 to July 2011. Quetzaltenango (bolded) surveillance was used for the base rate as well as the healthcare utilization survey. (DOCX) [file pone.0056882.s003.docx]

**Table S3**- Rates (per 1,000) of hospitalized and non-hospitalized influenza-associated pneumonia in Guatemala among children < 5 years of age, August 2009 to July 2011. Quetzaltenango (bolded) surveillance was used for the base rate as well as the healthcare utilization survey.

| **Department** | **Adjustment for Risk Factor prevalence and DHS Healthcare-seeking for ARI compared with base-rate province^1^** | **Percent of pneumonia cases hospitalized from HUS^2^** | **Hospitalized Rate (per 1,000) Aug 2009-July 2010^3^** | **Non-Hospitalized Rate (per 1,000) Aug 2009-July 2010^3^** | **Hospitalized Rate (per 1,000) Aug 2010-July 2011^4^** | **Non-Hospitalized Rate (per 1,000) Aug 2010-July 2011^4^** |
| --- | --- | --- | --- | --- | --- | --- |
| Guatemala | 0.86 | 0.38 | 0.54 | 0.89 | 0.52 | 0.85 |
| El Progreso | 0.78 | 0.31 | 0.49 | 1.08 | 0.47 | 1.03 |
| Sacatepequez | 1.05 | 0.38 | 0.66 | 1.06 | 0.63 | 1.01 |
| Chimaltenango | 1.14 | 0.35 | 0.72 | 1.33 | 0.69 | 1.27 |
| Escuintla | 0.93 | 0.35 | 0.58 | 1.09 | 0.56 | 1.04 |
| Solola | 1.16 | 0.34 | 0.73 | 1.41 | 0.70 | 1.35 |
| Totonicapan | 1.30 | 0.38 | 0.82 | 1.34 | 0.78 | 1.28 |
| Suchitepequez | 0.88 | 0.33 | 0.56 | 1.12 | 0.53 | 1.07 |
| Retalhuleu | 1.00 | 0.38 | 0.63 | 1.01 | 0.60 | 0.97 |
| San Marcos | 0.87 | 0.28 | 0.55 | 1.37 | 0.52 | 1.31 |
| Huehuetenango | 1.22 | 0.36 | 0.77 | 1.35 | 0.73 | 1.29 |
| Quiche | 1.32 | 0.36 | 0.83 | 1.47 | 0.80 | 1.41 |
| Baja Verapaz | 1.30 | 0.46 | 0.82 | 0.97 | 0.79 | 0.93 |
| Alta Verapaz | 1.02 | 0.34 | 0.64 | 1.23 | 0.61 | 1.18 |
| Peten | 1.09 | 0.37 | 0.69 | 1.17 | 0.66 | 1.12 |
| Izabal | 0.93 | 0.31 | 0.59 | 1.29 | 0.56 | 1.24 |
| Zacapa | 0.94 | 0.32 | 0.59 | 1.27 | 0.57 | 1.21 |
| Chiquimula | 1.20 | 0.36 | 0.76 | 1.35 | 0.73 | 1.29 |
| Jalapa | 1.17 | 0.35 | 0.74 | 1.38 | 0.71 | 1.32 |
| Jutiapa | 1.01 | 0.32 | 0.64 | 1.32 | 0.61 | 1.27 |
| **Quetzaltenango** | **1.00** | **0.40** | **0.63** | **0.95** | **0.60** | **0.91** |
| Santa Rosa | 0.84 | 0.29 | 0.53 | 1.29 | 0.51 | 1.23 |

1 This adjustment factor is based on 5 risk factors for ALRI and healthcare-seeking behaviors, adjusting the rate of the base province in bold to the other provinces. (${Adj}_{Y}$ from Equation 2a). Data available from National Survey of Maternal and Child Health 2008-2009 (Encuesta Nacional de Salud Materno-Infantil [ENSMI] 2008-2009). ARI is acute respiratory illness.

2 This adjustment factor is used to estimate the rate of non-hospitalized cases assumed to be of the same severity as hospitalized cases. HUS is Healthcare Utilization Survey. (${HUS}_{Y}$ from Equation 4).

3 Quetzaltenango base rate for children < 5 years in August 2009 to July 2010 is 8.65 per 1,000

4 Quetzaltenango base rate for children < 5 years in August 2010 to July 2011 is 12.14 per 1,000
