# Supplementary material for: Estimation of the National Disease Burden of Influenza-Associated Severe Acute Respiratory Illness in Kenya and Guatemala: A Novel Methodology
Source: PLoS One. 2013 Feb 27;8(2):e56882. doi: 10.1371/journal.pone.0056882 (PMC3584100; doi:10.1371/journal.pone.0056882)
Supplement: Table S4 — Rates (per 1,000) of hospitalized and non-hospitalized influenza-associated pneumonia in Guatemala among persons ≥5 years of age, August 2009 to July 2011. Quetzaltenango (bolded) surveillance was used for the base rate as well as the healthcare utilization survey. (DOCX) [file pone.0056882.s004.docx]

**Table S4**- Rates (per 1,000) of hospitalized and non-hospitalized influenza-associated pneumonia in Guatemala among persons ≥ 5 years of age, August 2009 to July 2011. Quetzaltenango (bolded) surveillance was used for the base rate as well as the healthcare utilization survey.

| **Department** | **Adjustment for Risk Factor prevalence and DHS Healthcare-seeking for ARI compared with base-rate province^1^** | **Percent of pneumonia cases hospitalized from HUS^2^** | **Hospitalized Rate (per 1,000) Aug 2009-July 2010^3^** | **Non-Hospitalized Rate (per 1,000) Aug 2009-July 2010^3^** | **Hospitalized Rate (per 1,000) Aug 2010-July 2011^4^** | **Non-Hospitalized Rate (per 1,000) Aug 2010-July 2011^4^** |
| --- | --- | --- | --- | --- | --- | --- |
| Guatemala | 0.63 | 0.15 | 0.12 | 3.85 | 0.04 | 0.23 |
| El Progreso | 0.66 | 0.13 | 0.13 | 4.89 | 0.04 | 0.30 |
| Sacatepequez | 0.86 | 0.16 | 0.17 | 5.21 | 0.06 | 0.31 |
| Chimaltenango | 0.95 | 0.14 | 0.19 | 6.30 | 0.06 | 0.38 |
| Escuintla | 0.76 | 0.14 | 0.15 | 5.06 | 0.05 | 0.31 |
| Solola | 1.13 | 0.14 | 0.22 | 7.66 | 0.08 | 0.47 |
| Totonicapan | 1.20 | 0.16 | 0.23 | 7.32 | 0.08 | 0.44 |
| Suchitepequez | 0.89 | 0.14 | 0.17 | 6.27 | 0.06 | 0.38 |
| Retalhuleu | 1.02 | 0.16 | 0.20 | 6.14 | 0.07 | 0.37 |
| San Marcos | 0.86 | 0.12 | 0.17 | 7.02 | 0.06 | 0.44 |
| Huehuetenango | 1.16 | 0.15 | 0.23 | 7.42 | 0.08 | 0.45 |
| Quiche | 1.25 | 0.15 | 0.24 | 8.05 | 0.08 | 0.49 |
| Baja Verapaz | 1.40 | 0.19 | 0.27 | 7.03 | 0.09 | 0.41 |
| Alta Verapaz | 1.15 | 0.14 | 0.22 | 7.81 | 0.08 | 0.48 |
| Peten | 1.09 | 0.15 | 0.21 | 6.79 | 0.07 | 0.41 |
| Izabal | 0.79 | 0.13 | 0.15 | 5.93 | 0.05 | 0.37 |
| Zacapa | 0.77 | 0.13 | 0.15 | 5.64 | 0.05 | 0.35 |
| Chiquimula | 0.99 | 0.15 | 0.19 | 6.41 | 0.07 | 0.39 |
| Jalapa | 0.98 | 0.14 | 0.19 | 6.55 | 0.07 | 0.40 |
| Jutiapa | 0.85 | 0.13 | 0.17 | 6.13 | 0.06 | 0.38 |
| **Quetzaltenango** | **1.00** | **0.16** | **0.20** | **5.78** | **0.07** | **0.34** |
| Santa Rosa | 0.72 | 0.12 | 0.14 | 5.80 | 0.05 | 0.36 |

1 This adjustment factor is based on 2 risk factors for ALRI and healthcare-seeking behaviors, adjusting the rate of the base province in bold to the other provinces. (${Adj}_{Y}$ from Equation 2a). Data available from National Survey of Maternal and Child Health 2008-2009 (Encuesta Nacional de Salud Materno-Infantil [ENSMI] 2008-2009). ARI is acute respiratory illness.

2 This adjustment factor is used to estimate the rate of non-hospitalized cases assumed to be of the same severity as hospitalized cases. HUS is Healthcare Utilization Survey. (${HUS}_{Y}$ from Equation 4).

3 Quetzaltenango base rate for older persons in August 2009 to July 2010 is 0.86 per 1,000

4 Quetzaltenango base rate for older persons in August 2010 to July 2011 is 0.89 per 1,000
