# Supplementary material for: Estimation of the National Disease Burden of Influenza-Associated Severe Acute Respiratory Illness in Kenya and Guatemala: A Novel Methodology
Source: PLoS One. 2013 Feb 27;8(2):e56882. doi: 10.1371/journal.pone.0056882 (PMC3584100; doi:10.1371/journal.pone.0056882)
Supplement: Table S5 — Rates and 95% confidence limits (per 1,000) of hospitalized and non-hospitalized influenza-associated severe acute respiratory illness (SARI) in Kenyan children <5 years of age, August 2009 to July 2010. Kilifi, Coast province (bolded) surveillance was used for the base rate. (DOCX) [file pone.0056882.s005.docx]

**Table S5**- Rates and 95% confidence limits (per 1,000) of hospitalized and non-hospitalized influenza-associated severe acute respiratory illness (SARI) in Kenyan children < 5 years of age, August 2009 to July 2010. Kilifi, Coast province (bolded) surveillance was used for the base rate.

| **Department** | **Adjustment for Risk Factor prevalence and DHS Healthcare-seeking for ARI compared with base-rate province^1^** | **Percent of pneumonia cases hospitalized from HUS^2^** | **Hospitalized Rate (per 1,000) Aug 2009-July 2010^3^** | **(95% Confidence Limit)** | **Non-Hospitalized Rate (per 1,000) Aug 2009-July 2010^3^** | **(95% Confidence Limit)** |
| --- | --- | --- | --- | --- | --- | --- |
| Central | 0.89 | 0.38 | 3.3 | (1.7 – 5.2) | 5.2 | (3.8 – 7.5) |
| **Coast** | **1.00** | **0.48** | **2.9** | **(1.8 – 4.0)** | **3.1** | **(1.8 – 5.4)** |
| Eastern | 1.05 | 0.45 | 1.0 | (0.0 – 3.5) | 1.3 | (0.0 – 4.4) |
| Nairobi | 0.58 | 0.58 | 0.6 | (0.2 – 1.1) | 0.4 | (0.2 – 0.9) |
| North Eastern | 1.18 | 0.52 | 3.5 | (1.4 – 7.7) | 3.2 | (1.6 – 4.9) |
| Nyanza | 1.20 | 0.46 | 2.3 | (1.6 – 3.4) | 2.7 | (2.0 – 4.0) |
| Rift Valley | 1.08 | 0.49 | 4.7 | (3.1 – 6.9) | 4.9 | (3.4 – 7.8) |
| Western | 0.74 | 0.39 | 2.3 | (1.4 – 3.9) | 3.7 | (2.8 – 5.1) |

1 This adjustment factor is based on 6 risk factors for SARI and healthcare-seeking behaviors, adjusting the rate of the base province in bold to the other provinces. (${Adj}_{Y}$ from Equation 2a). DHS is Demographic and Health Survey. ARI is acute respiratory illness.

2 This adjustment factor is used to estimate the rate of non-hospitalized cases assumed to be of the same severity as hospitalized cases. HUS is Healthcare Utilization Survey. (${HUS}_{Y}$ from Equation 4).

3 Kilifi (Coast province) base rate for children < 5 years of age in August 2009 to July 2010 is 27.32 per 1,000
