# Supplementary material for: Estimation of the National Disease Burden of Influenza-Associated Severe Acute Respiratory Illness in Kenya and Guatemala: A Novel Methodology
Source: PLoS One. 2013 Feb 27;8(2):e56882. doi: 10.1371/journal.pone.0056882 (PMC3584100; doi:10.1371/journal.pone.0056882)
Supplement: Appendix S1 — Equations used in calculation of annual number of cases of influenza-associated severe acute respiratory infection (SARI) cases. (DOCX) [file pone.0056882.s006.docx]

**Appendix S1**. Equations used in calculation of annual number of cases of influenza-associated severe acute respiratory infection (SARI) cases.

**Equation 1: Incidence rate of hospitalized SARI in base province**

$$I_{B}=\frac{{SARI}_{B}}{{Pop}_{B}}$$

Where:

$I_{B}$ = Base rate of hospitalized SARI

${SARI}_{B}$ = Number of total cases meeting SARI case definition hospitalized in base province per year

${Pop}_{B}$ = Population of surveillance catchment area

**Equation 2a: Calculation of adjustment for risk factors at the provincial level for base rate hospitalized SARI**

$${Adj}_{Y}=\left( 1+\sum_{i} \left( P_{i,Y}-P_{i,B} \right)\times\left( {RR}_{i}-1 \right) \right)$$

Where:

${Adj}_{Y}$ = Adjustment factor for province Y for risk factors of SARI

$P_{i,Y}$ = Prevalence of risk factor *i* in province Y

$P_{i,B}$= Prevalence of risk factor *i* in base province

${RR}_{i}$ = Relative risk of SARI due to risk factor *i*

**Equation 2b: Rate of hospitalized SARI in province after adjustment for risk factors and healthcare-seeking behavior**

$$I_{H,Y}=I_{B} \times{Adj}_{Y} \times\frac{{DHS}_{Y}}{{DHS}_{B}}$$

Where:

$I_{H,Y}$ = Incidence of hospitalized SARI in province Y

${DHS}_{Y}$ = Proportion of ARI cases seeking care in province Y (from DHS)

${DHS}_{B}$ = Proportion of ARI cases seeking care in base province (from DHS)

**Equation 3: Rate of influenza-associated hospitalized SARI in province**

$${IF}_{H,Y}=I_{H,Y} \times F_{Y}$$

Where:

${IF}_{H,Y}$= Incidence of hospitalized influenza-associated SARI in province Y

$F_{Y}$ = Proportion of pneumonia due to influenza

**Equation 4: Rate of influenza-associated non-hospitalized SARI in province**

$${IF}_{NH,Y}=\left( {IF}_{H,Y} \times\frac{1}{{HUS}_{Y}} \right)- {IF}_{H,Y}$$

Where:

${IF}_{NH,Y}$= Incidence of non-hospitalized influenza-associated SARI in province Y

${HUS}_{Y}$ = Proportion of all SARI cases that are hospitalized in province Y

$${HUS}_{Y}={HUS}_{B}\times\frac{{DHS}_{Y}}{{DHS}_{B}}$$

Where:

${HUS}_{B}$ = Proportion of all SARI cases that are hospitalized in the base province

**Equation 5: Number of influenza-associated hospitalized and non-hospitalized SARI cases in province**

$${NF}_{H,Y}={IF}_{H,Y} \times P{op}_{Y}$$

$${NF}_{NH,Y}={IF}_{NH,Y} \times P{op}_{Y}$$

Where:

${NF}_{H,Y}$= Number of hospitalized influenza-associated SARI cases in province Y

${NF}_{NH,Y}$= Number of non-hospitalized influenza-associated SARI cases in province Y

$P{op}_{Y}$= Population in province Y
