# Supplementary material for: Estimation of the National Disease Burden of Influenza-Associated Severe Acute Respiratory Illness in Kenya and Guatemala: A Novel Methodology
Source: PLoS One. 2013 Feb 27;8(2):e56882. doi: 10.1371/journal.pone.0056882 (PMC3584100; doi:10.1371/journal.pone.0056882)
Supplement: Appendix S2 — Equation used to calculate HIV prevalence in children. PMTCT is Prevention of Mother to Child Transmission. (DOCX) [file pone.0056882.s007.docx]

**Appendix S2**. Equation used to calculate HIV prevalence in children. PMTCT is Prevention of Mother to Child Transmission.

**HIV Prev_Child_ = (Prev_PMTCT_ * TR_PMTCT_) + (Prev _Non-PMTCT_ * TR_NonPMTCT_) + ((Prev _HIV+_ -(Prev_PMTCT_ * TR_PMTCT_) - (Prev _Non-PMTCT_ * TR_NonPMTCT_))* TR_bf_)**

Where:

HIV Prev_Child_ = HIV prevalence in children < 5

Prev_PMTCT_ = Prevalence of HIV+ pregnant women participating in PMTCT (Based on prevalence of HIV+ women receiving ante-natal care, DHS, 2009)

TR_PMTCT_ = Transmission rate from mothers receiving PMTCT (Hellen Muttai, personal communication, July 22, 2011)

Prev _Non-PMTCT_ = Prevalence of HIV+ pregnant women not participating in PMTCT; (1- Prev_PMTCT_)

TR _Non-PMTCT_ = Transmission rate from mothers not participating in PMTCT (*46*)

Prev _HIV+_ = Prevalence of HIV+ pregnant women (*47*)

TR_bf_ = Transmission rate from breastfeeding (*48*)
